# Dataset of gridded time series of monthly air temperature (min, max, mean) and atmospheric precipitation for Ukraine covering the period of 1946–2020

**DOI:** 10.1016/j.dib.2022.108553

**Published:** 2022-08-24

**Authors:** Volodymyr Osadchyi, Olesya Skrynyk, Liudmyla Palamarchuk, Oleg Skrynyk, Valeriy Osypov, Dmytro Oshurok, Vladyslav Sidenko

**Affiliations:** a Ukrainian Hydrometeorological Institute, Kyiv, Ukraine; b National University of Life and Environmental Sciences of Ukraine, Kyiv, Ukraine

**Keywords:** Gridded climate data, Monthly min/max/mean air temperature, Monthly atmospheric precipitation, Ukraine

## Abstract

In the paper, we present a dataset of long gridded time series of monthly minimum, maximum and mean air temperature and atmospheric precipitation for Ukraine (one of the largest European countries), covering the period of 1946–2020. The dataset was built through the thorough historical climate data processing, which included all mandatory steps: data rescue/digitization of missing values and/or periods in station time series from paper sources, their quality control (QC) and homogenization, and finally interpolation on 0.1^o^ × 0.1^o^ grid. The station data comprised monthly values of 178 stations for air temperature (for each of three parameters) and 224 stations for atmospheric precipitation. The quality assurance and homogenization were performed by means of the widely used homogenization software HOMER (HOMogEnization in R), while the well-known interpolation software MISH (Meteorological Interpolation based on Surface Homogenized data basis) was used to perform the gridding. The dataset has the potential to be used in a wide variety of applications, such as regional climate change and variability studies, assessment of other climate gridded products (e.g., reanalysis or analysis), global/regional climate model output verification, etc.

**Specifications Table**

**Table:** 

Subject	Climatology
Specific subject area	Climate data processing, Climate change
Type of data	Gridded time series. For user convenience, they are provided in data files of two different types: csv-files ser-files (plain ASCII files with extension “.ser”, which are output of the MISH software) Except the data files, metadata files with IDs and geographical coordinates of grid points are also provided: dat-files
How the data were acquired	Station monthly time series for the period of 1946–1965 were digitized from paper sources (officially published handbooks/reports [1], [2], [3]). The station monthly data for 1966–2020 were calculated from corresponding daily values, which were provided by the Central Geophysical Observatory (an observation institution of the Ukrainian Weather Service) in digital form. Subdaily data for 2015–2020 from the temporary occupied territory (the Crimea and parts of Donetsk and Lugansk regions) were downloaded from freely available internet resources [4,5] and then used to calculate corresponding daily and monthly values. QC and homogenization of the station time series were performed by means of the HOMER software [6]. Gridding of the homogenized station data was performed by means of the MISH software [7].
Data format	Calculated (Quality controlled/Homogenized/Interpolated) Data.
Description of data collection	Data rescue/digitization of historical station time series was performed manually. All calculations (QC, homogenization, gridding) were performed on a personal computer.
Data source location	Monthly data for 1946–1965 Institution: Ukrainian Hydrometeorological Institute (UHMI) City/Town/Region: Kyiv Country: Ukraine Daily data for 1966–2020 Institution: Central Geophysical Observatory City/Town/Region: Kyiv Country: Ukraine Subdaily data for 2015–2020 from the temporary occupied Ukrainian territory: http://pogodaiklimat.ru http://meteomanz.com/
Data accessibility	Repository name: Data Repository of the Ukrainian Hydrometeorological Institute Data identification number: https://doi.org/10.15407/uhmi.report.02 Direct URL to data: https://uhmi.org.ua/eng/data_repo/month/ Instructions for accessing these data: The data are freely available for scientific purposes without any registration.

## Value of the Data

• The dataset of gridded climatological time series is useful because it covers a large part of the Eastern Europe over a fairly long period (75 years). The territory of Ukraine has been generally overlooked, in a sense of free and high quality observed climate data availability for international scientific researches/projects/studies.
• In addition, the dataset is usable and valuable because it was built based on the most complete empirical basis (i.e. almost all air temperature and atmospheric precipitation measurements, which were performed on the territory of Ukraine over 1946–2020, were used to construct the dataset) and following the thorough and well-approved historical climate data processing pathway.
• Researchers (climatologists, meteorologists, hydrologists, hydrochemists, ecologists, etc) can utilize the data to perform scientific studies related to the regional climate, its change and variability. Climate modelers can exploit the data collection to evaluate climate model outputs.

## 1 Data Description

The dataset, which can be accessed at the Data Repository of the Ukrainian Hydrometeorological Inistitute [8], is structured according to four climate parameters considered, TN (minimum air temperature), TX (maximum air temperature), TG (mean air temperature) and RR (atmospheric precipitation). Consequently, the notations TN, TX, TG and RR are used as names of separate directories, which contain corresponding data and metadata files. Each directory includes three files: *MonthlyGrid.csv, MonthlyGrid.ser* and *predtandfila.dat*.

**Fig 1:**
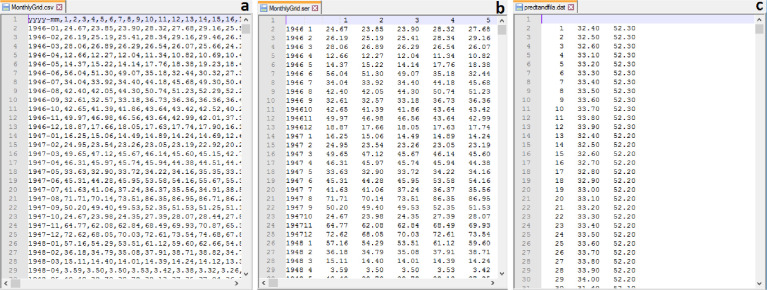
Examples of fragments of data and metadata files from the dataset: (a) *MonthlyGrid.csv*, monthly gridded time series in the csv-format, (b) *MonthlyGrid.ser*, monthly gridded time series in the ser-format (MISH output), (c) *predtandfila.dat*, a list of grid point IDs and geographical coordinates.
• *MonthlyGrid.ser* (Fig. 1b) is a data file containing the same gridded data in the ser-format, which is used in the MISH software for the output files. In fact, it is a plain ASCII file, which can be opened in any text editor like Notepad etc. This file is convenient to read in Fortran.
• *predtandfila.dat* (Fig. 1c) is a metadata file containing geographical coordinates (longitudes and latitudes) of all grid points located on the territory of Ukraine. First column, containing IDs of grid points, is followed by corresponding values of longitudes and latitudes.

• *MonthlyGrid.csv* (Fig. 1a) is a data file, which contains the gridded time series in the csv-format. Such format is convenient when using modern script languages, like R or Python, to read the data for further analysis.

As can be seen from Fig. 1(a, b), the formats of the records in both data files, *MonthlyGrid.csv* and *MonthlyGrid.ser*, are quite similar. The first record/line is a header, which is followed by data lines for each year-month of the period. The header contains IDs of grid points. The data lines contain one or two values (depending on the data file) of year and month and then values of a climate parameter are listed for each grid point. In Fortran90, the data from *MonthlyGrid.ser* can be read using the following format statement: format (i4, i2, 7385f8.2), where 7385 is the number of grid points covering the territory of Ukraine.

Units used for monthly mean air temperature data and monthly accumulated sum of atmospheric precipitation are Celsius degrees (°C) and millimeters (mm), respectively.

## 2 Experimental Design, Materials and Methods

### 2.1 Data Rescue/Digitization, Compilation Of Monthly Raw Station Time Series, Their QC And Homogenization

The pre-interpolation part of our method included the following steps.

**Fig. 2:**
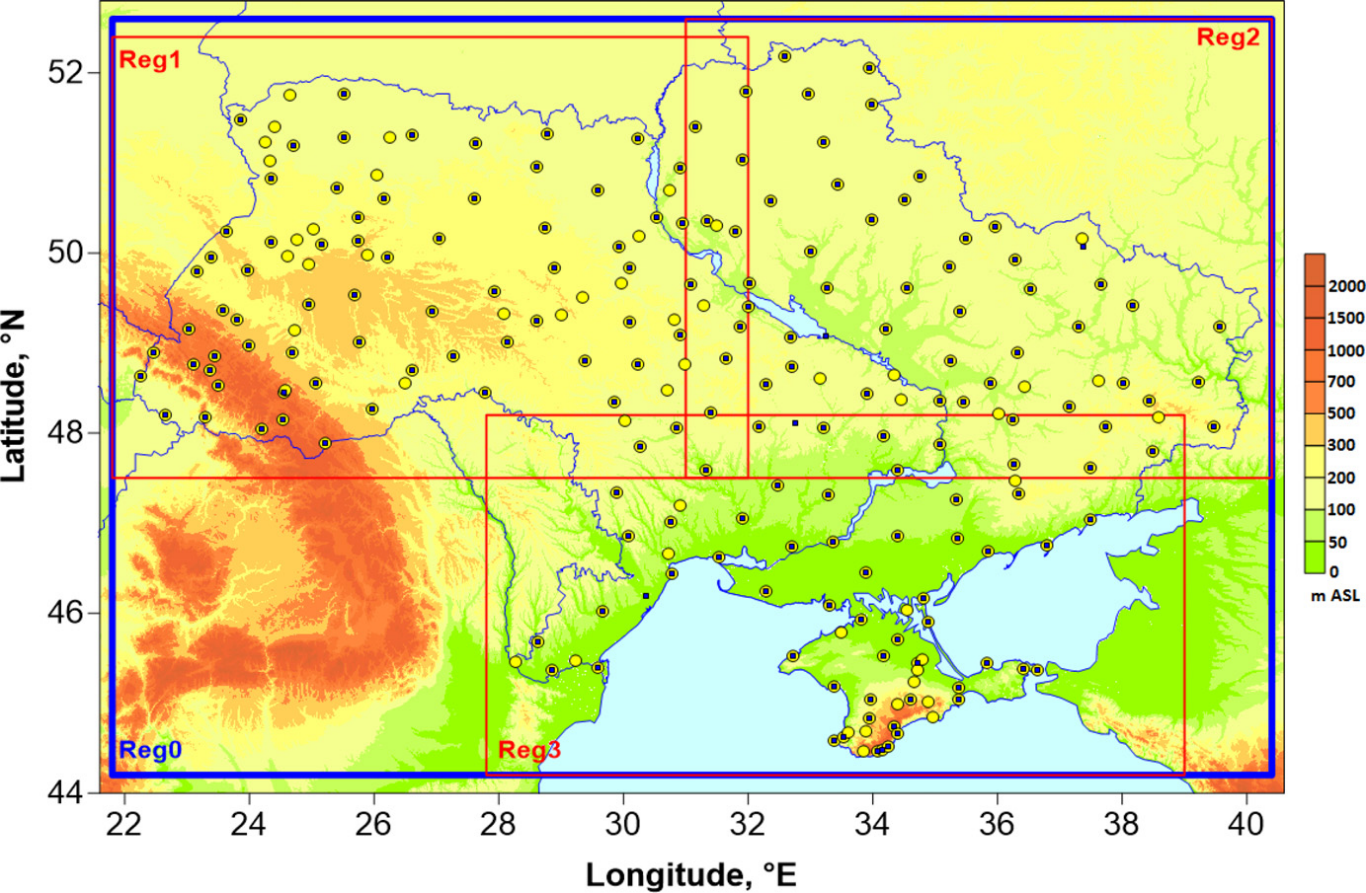
The domain under study with relief (note, no coordinate projection was used in the map). Blue squares display locations of 178 climatological stations where air temperature data were collected, while 224 yellow circles show stations with atmospheric precipitation time series. Red frames (Reg1, Reg2 and Reg3) denote three overlapping regions where the gridding was performed separately. The common blue frame, Reg0, combined the results for Reg1, Reg2 and Reg3 (harmonization/averaging was applied in the overlapping areas).
• For RR, a total number of stations was 224 (see Fig 2). This quantity includes 174 stations, which were used for air temperature, and extra 50 hydrological posts, where atmospheric precipitation are also measured in addition to hydrological variables. The remaining four climatological stations were excluded because of long missing periods in their precipitation time series. By using data from the posts, we tried to collect and involve all available precipitation measurements keeping in mind larger spatial heterogeneity of precipitation fields compared to air temperature, even on the monthly time scale. A complete list of the stations and posts is provided as a supplemental file (SF1).
• TN, TX, TG and RR monthly data for the period of 1946–1965 were obtained through data rescue activities aiming to preserve and digitize valuable climatological information from paper sources, i.e. handbooks and reports, which were officially published in Ukraine [1], [2], [3]. The station monthly data for 1966–2020 were calculated from corresponding daily values provided by the Central Geophysical Observatory (an observation institution of the Ukrainian Weather Service) in digital form. The World Meteorological Organization (WMO) “3 and 5” rule [9] was applied to calculate monthly data from daily values. Metadata, mainly dates of station relocations, which are important for a homogenization procedure, were also collected from historical descriptions of the stations.
• Unfortunately, Ukraine has lost control of a certain number of climatological stations and hydrological posts (26 of 178 and 12 of 50, respectively; located in the Crimea and Eastern parts of Lugansk and Donetsk regions) since 2015. Data for the last six years from these stations are not directly available for Ukrainian scientists. However, in order to have a complete dataset for the period 1946–2020, we tried to find such data on the Internet. Fortunately, subdaily (3-hourly) data for 20 stations (mainly, from the Crimea) covering 2015–2020 were found and downloaded from [4,5] and then used to calculate corresponding daily and monthly values. Data from the other inaccessible climatological stations or hydrological posts for period of 2015–2020 were assumed to be missing. Calculation of daily mean air temperature and daily sum of atmospheric precipitation from the subdaily data is rather straightforward and was performed according to definitions of these parameters. Daily min/max air temperature was calculated as a minimal/maximal value of eight measurements for a particular day. Such approach can introduce some errors to TN and TX data, but in our case, the raw errored data are still better than missing values. Therefore, the final TN and TX interpolated data for the Crimea for period 2015–2020 should be treated/used with caution.
• QC and homogenization of the compiled station time series were performed by means of the HOMER v2.6 software [6]. HOMER was developed under the umbrella of the COST Action ES0601. It integrates the parts of different relative QC and homogenization algorithms, tested and validated *via* benchmarking [10].
• The air temperature time series were firstly homogenized for the period 1946–2014 (TG) [11] and 1946–2015 (TN and TX) [12]. However, after compiling the raw station time series for the extended period, 1946–2020, the QC and homogenization procedures were repeated for temperature time series one more time. Similar to [11,12] we applied an interactive two-cycle homogenization process, as suggested by HOMER authors [13]. The first cycle was performed on the raw data. The second run was applied to the homogenized data obtained on the previous step. Testing for inhomogeneities for each time series was made using 15 reference/neighboring stations, which were the most highly correlated to each candidate station.
• RR time series were quality controlled and homogenized based on the similar algorithm as for air temperature data. However, unlike HOMER's additive correction option, which is used for air temperature time series, multiplicative correction was selected when homogenizing atmospheric precipitation data.

• For TN, TX and TG variables, measurements conducted at 178 climatological stations (see Fig. 2) were processed. These 178 stations constitute the entire modern Ukrainian observational meteorological network. The beginning of the period under study, 1946–2020, was chosen due to an extremely large number of missing values during the World War II, which prevented from performing relative homogenization for any longer (extended to earlier dates) period.

### 2.2 Gridding

In order to grid the homogenized station data, we used the MISH v1.03 software [7], which was developed specifically for meteorological/climatological purposes. MISH is a hybrid gridding approach, which along with deterministic interpolation algorithms also uses geospatial stochastic modeling. However, compared to the latter, MISH takes advantage to utilize valuable climatological information contained inside long station time series in order to define statistical parameters (weighting factors) in the interpolation formulae.

The software consists of two parts, modeling and interpolation. The first part is used to define statistical dependences between a climate parameter to be interpolated and a set of supplementary deterministic predictors, such as geophysical or terrain characteristics (a number and type of such predictors are defined by a user). The following supplementary predictors are usually used in MISH applications: height above sea level, AURELHY (Analyse Utilisant le RELief pour les besoins del’ HYdrométéorologie) principle components [14] that completely describe local topography, the Euclidean distance to the coastline etc. The results of the modelling part are then used during the interpolation phase. The modeling part is run only once (for the whole period under study), while interpolation is performed for each year-month (time step) individually.

In order to construct our dataset, a grid with spatial resolution of 0.1^o^ × 0.1^o^ was defined for the whole territory of Ukraine (Reg0 in Fig. 2). Due to some MISH limitations on a maximum number of grid points, it was impossible to perform calculations based on a single run of the MISH software. Thus, the entire territory of Ukraine was split into three overlapping regions (Reg1, Reg2 and Reg3 in Fig. 2), where the interpolation was performed separately. Interpolation grids in these regions were defined as parts of the common grid introduced in Reg0. As additional deterministic predictors in Reg1 and Reg2, we used elevation above sea level and first fifteen AURELHY components, which were calculated based on Digital Elevation Model data GTOPO30 [15]. In Reg3 apart from the mentioned above predictors, the distance to the coastline was also used (Table 1).

**Table 1:** Deterministic predictors used in the modeling part of the MISH algorithm for each interpolation region.

	Predictors		
Region	Height ASL	AURELHY principle components (first 15 values)	Distance to the coastline
Reg1	+	+	-
Reg2	+	+	-
Reg3	+	+	+

After obtaining results for Reg1–3, a harmonization procedure in overlapping areas was applied in order to avoid possible discontinuities on the edges of the regions when building the dataset for the whole Ukraine. The harmonization was performed by averaging values from different regions at the same grid points and the same time steps (year-month). Finally, we removed from the harmonized dataset all gridded time series, which belong to grid points beyond the territory of Ukraine. It is a necessary step since we do not use station data from neighboring countries and, consequently, interpolation in these neighboring areas will not reflect real spatial distribution of the meteorological parameters.

Assessments of the interpolation accuracy, obtained based on a cross-validation procedure, can be found in [16].

## Ethics statements

This dataset does not include any human subjects, animal experiment, or social media platforms.

## CRediT authorship contribution statement

**Volodymyr Osadchyi:** Conceptualization, Supervision, Writing – review & editing. **Olesya Skrynyk:** Methodology, Data curation, Formal analysis, Writing – original draft. **Liudmyla Palamarchuk:** Methodology, Data curation, Formal analysis, Writing – original draft. **Oleg Skrynyk:** Methodology, Formal analysis, Software, Writing – original draft, Writing – review & editing. **Valeriy Osypov:** Data curation, Software, Writing – review & editing. **Dmytro Oshurok:** Data curation, Visualization, Writing – review & editing. **Vladyslav Sidenko:** Data curation, Visualization, Writing – review & editing.

## Declaration of Competing Interest

The authors declare that they have no known competing financial interests or personal relationships that could have appeared to influence the work reported in this paper.

## Data Availability

Ukrainian gridded monthly air temperature (min, max, mean) and atmospheric precipitation data (1946–2020) (Original data) (Data Repository of Ukrainian Hydrometeorological Institute).

## Appendix Supplementary materials

Image, application 1

## Footnotes

Supplementary material associated with this article can be found, in the online version, at doi:10.1016/j.dib.2022.108553.

## References

[1] Ukrainian Research Hydrometeorological Institute (1953).

[2] Kiev Hydrometeorological Observatory (1971).

[3] Kiev Hydrometeorological Observatory (1971).

[4] Archives of meteorological data. http://pogodaiklimat.ru, 2021. Accessed December 10, 2021.

[5] Meteorological data. http://meteomanz.com/, 2005. Accessed July 22, 2022.

[6] Mestre O., Domonkos P., Picard F., Auer I., Robin S., Lebarbier E., Bohm R., Aguilar E., Guijarro J., Vertachnik G., Klancar M., Dubuisson B., Stepanek P. (2013). HOMER: a homogenization software - methods and applications. Idojaras (Q. J. Hung. Meteorol. Serv.).

[7] Szentimrey T., Bihari Z. (2014).

[8] Osadchyi V., Skrynyk O.A., Palamarchuk L., Skrynyk O.Y., Osypov V., Oshurok D., Sidenko V. (2022). UHMI Data Repos..

[9] WMO (1989). Calculation of Monthly and Annual 30-Years Standard Normals.

[10] Venema V., Mestre O., Aguilar E., Auer I., Guijarro J.A., Domonkos P., Vertacnik G., Szentimrey T., Stepanek P., Zahradnicek P., Viarre J., Muller-Westermeier G., Lakatos M., Williams C.N., Menne M., Lindau R., Rasol D., Rustemeier E., Kolokythas K., Marinova T., Andresen L., Acquaotta F., Fratianni S., Cheval S., Klancar M., Brunetti M., Gruber C., Duran M.P., Likso T., Esteban P., Brandsma T. (2012). Benchmarking monthly homogenization algorithms. Clim. Past.

[11] Osadchyi V., Skrynyk O.A., Radchenko R., Skrynyk O.Y. (2018). Homogenization of Ukrainian air temperature time series. Int. J. Climatol..

[12] Skrynyk O.Y., Aguilar E., Skrynyk O.A., Sidenko V., Boichuk D., Osadchyi V. (2019). Quality control and homogenization of monthly extreme air temperature of Ukraine. Int. J. Climatol..

[13] O. Mestre, E. Aguilar, HOME_R. fast documentation. HOMER training school, 36 pp. 2011. Available at: http://www.c3.urv.cat/data/HOME_R.pdf. Accessed July 22, 2022.

[14] Benichou P., Breton O.Le (1987). Deuxiиmes Journues Hydrologiques de l'ORSTOM а Montpellier (Colloques et Seminaires).

[15] USGS. Global 30 arc-second elevation (GTOPO30) doi:10.5066/F7DF6PQS. Accessed July 22, 2022.

[16] Skrynyk O.A., Osadchyi V.I., Szentimrey T., Bihari Z., Sidenko V.P., Oshurok D.O., Boichuk D.O., Skrynyk O.Y. (2020). Spatial interpolation of climatological data with relief and physicogeographical peculiarities of the territory of Ukraine taken into account. Ukr. Geogr. J..

