# Supplementary material for: Dataset of gridded time series of monthly air temperature (min, max, mean) and atmospheric precipitation for Ukraine covering the period of 1946–2020
Source: Data Brief. 2022 Aug 24;44:108553. doi: 10.1016/j.dib.2022.108553 (PMC9469657; doi:10.1016/j.dib.2022.108553)
Supplement: Supplementary file 1 [file mmc1.docx]

| # | WMO code | Station/post name | Longitude | Latitude | Altitude | Koeppen-Geiger  climate type |
| --- | --- | --- | --- | --- | --- | --- |
| Meteorological stations | | | | | | |
| 1 | 33075 | Lyubeshiv | 25.5167 | 51.7667 | 149.0 | Dfb |
| 2 | 33067 | Svityaz | 23.8492 | 51.4817 | 164.0 | Dfb |
| 3 | - | Manevychi | 25.5167 | 51.2833 | 201.0 | Dfb |
| 4 | 33173 | Kovel | 24.7067 | 51.1978 | 173.0 | Dfb |
| 5 | 33177 | Volodymyr-Volynskyi | 24.3500 | 50.8333 | 192.8 | Dfb |
| 6 | 33187 | Lutsk | 25.3956 | 50.7208 | 195.3 | Dfb |
| 7 | 33088 | Sarny | 26.6167 | 51.3167 | 154.0 | Dfb |
| 8 | 33301 | Rivne_AMSC | 26.1528 | 50.6025 | 227.0 | Dfb |
| 9 | 33296 | Dubno | 25.7500 | 50.4000 | 198.0 | Dfb |
| 10 | 33287 | Rava-Ruska | 23.6272 | 50.2422 | 252.0 | Dfb |
| 11 | 33288 | Kamyanka-Buzka | 24.3500 | 50.1167 | 212.0 | Dfb |
| 12 | 33297 | Brody | 25.1500 | 50.1000 | 227.0 | Dfb |
| 13 | 33392 | Yavoriv | 23.3833 | 49.9500 | 254.0 | Dfb |
| 14 | 33391 | Mostyska | 23.1633 | 49.7914 | 232.0 | Dfb |
| 15 | 33393 | Lviv_AMSC | 23.9653 | 49.8075 | 319.0 | Dfb |
| 16 | 33398 | Drogobych_AMSC | 23.5669 | 49.3622 | 275.0 | Dfb |
| 17 | 33513 | Stryi | 23.8083 | 49.2578 | 302.0 | Dfb |
| 18 | 33511 | Turka | 23.0297 | 49.1503 | 594.0 | Dfb |
| 19 | 33516 | Slavske | 23.4500 | 48.8500 | 592.0 | Dfb |
| 20 | 33317 | Shepetivka | 27.0403 | 50.1619 | 277.0 | Dfb |
| 21 | 33421 | Yampil | 26.2050 | 49.9481 | 274.0 | Dfb |
| 22 | 33429 | Khmelnytsky_AMSC | 26.9375 | 49.3536 | 348.0 | Dfb |
| 23 | 33557 | Nova_Ushytsya | 27.2667 | 48.8500 | 292.0 | Dfb |
| 24 | 33548 | Kamyanets-Podilskyi | 26.6086 | 48.6933 | 217.0 | Dfb |
| 25 | 33299 | Kremenets | 25.7333 | 50.1333 | 259.0 | Dfb |
| 26 | 33415 | Ternopil_AMSC | 25.6911 | 49.5278 | 327.0 | Dfb |
| 27 | 33409 | Berezhany | 24.9500 | 49.4333 | 303.0 | Dfb |
| 28 | 33536 | Chortkiv | 25.7667 | 49.0167 | 320.0 | Dfb |
| 29 | 33524 | Dolyna | 23.9978 | 48.9769 | 470.0 | Dfb |
| 30 | 33526 | Ivano-Frankivsk_AMSC | 24.6889 | 48.8894 | 275.0 | Dfb |
| 31 | 33645 | Yaremche | 24.5500 | 48.4500 | 531.0 | Dfb |
| 32 | 33651 | Kolomyya | 25.0556 | 48.5489 | 295.0 | Dfb |
| 33 | 33646 | Pozhyzhevska | 24.5333 | 48.1500 | 1451.0 | Dfc |
| 34 | 33514 | Velykyi_Bereznyi | 22.4667 | 48.9000 | 205.0 | Dfb |
| 35 | 33517 | Nyzhni_Vorota | 23.1000 | 48.7667 | 496.0 | Dfb |
| 36 | 33518 | Nyzhnyi_Studenyi | 23.3667 | 48.7000 | 615.0 | Dfb |
| 37 | 33631 | Uzhgorod_AMSC | 22.2611 | 48.6333 | 113.0 | Dfb |
| 38 | 33633 | Mizhgirrya | 23.5044 | 48.5267 | 456.0 | Dfb |
| 39 | 33634 | Beregove | 22.6500 | 48.2000 | 113.0 | Dfb |
| 40 | 33638 | Khust | 23.2922 | 48.1786 | 164.0 | Dfb |
| 41 | 33647 | Rakhiv | 24.1981 | 48.0475 | 430.0 | Dfb |
| 42 | 33658 | Chernivtsi_AMSC | 25.9725 | 48.2664 | 242.0 | Dfb |
| 43 | 33657 | Selyatyn | 25.2167 | 47.8833 | 762.0 | Dfb |
| 44 | 33049 | Semenivka | 32.5833 | 52.1833 | 160.0 | Dfb |
| 45 | 33136 | Shchors | 31.9667 | 51.8000 | 127.0 | Dfb |
| 46 | 33146 | Pokoshychi | 32.9667 | 51.7667 | 193.0 | Dfb |
| 47 | 33135 | Chernigiv_AMSC | 31.1536 | 51.4067 | 140.0 | Dfb |
| 48 | 33246 | Nizhyn | 31.9000 | 51.0333 | 124.0 | Dfb |
| 49 | 33236 | Oster | 30.9000 | 50.9500 | 112.0 | Dfb |
| 50 | 33362 | Pryluky | 32.3636 | 50.5792 | 132.0 | Dfb |
| 51 | 33203 | Olevsk | 27.6333 | 51.2167 | 182.0 | Dfb |
| 52 | 33213 | Ovruch | 28.7836 | 51.3289 | 168.0 | Dfb |
| 53 | 33215 | Korosten | 28.6142 | 50.9550 | 185.0 | Dfb |
| 54 | 33312 | Novograd-Volynskyi | 27.6167 | 50.6000 | 216.0 | Dfb |
| 55 | 33325 | Zhytomyr_AMSC | 28.7328 | 50.2772 | 214.0 | Dfb |
| 56 | 33231 | Chornobyl | 30.2256 | 51.2664 | 123.0 | Dfb |
| 57 | 33228 | Teteriv | 29.5831 | 50.6928 | 132.0 | Dfb |
| 58 | 33345 | Kyiv | 30.5364 | 50.3922 | 166.0 | Dfb |
| 59 | 33347 | Boryspil | 30.9494 | 50.3358 | 121.0 | Dfb |
| 60 | 33354 | Baryshivka | 31.3375 | 50.3553 | 100.0 | Dfb |
| 61 | 33356 | Yagotyn | 31.8000 | 50.2333 | 126.0 | Dfb |
| 62 | 33339 | Fastiv | 29.9267 | 50.0692 | 208.0 | Dfb |
| 63 | 33464 | Bila_Tserkva | 30.1064 | 49.8286 | 179.0 | Dfb |
| 64 | 33466 | Myronivka | 31.0819 | 49.6456 | 151.0 | Dfb |
| 65 | 33484 | Zolotonosha | 32.0175 | 49.6672 | 94.0 | Dfb |
| 66 | 33487 | Cherkasy_AMSC | 32.0031 | 49.4092 | 106.0 | Dfb |
| 67 | 33581 | Zhashkiv | 30.1000 | 49.2333 | 235.0 | Dfb |
| 68 | 33593 | Smila | 31.8653 | 49.1844 | 126.0 | Dfb |
| 69 | 33605 | Chygyryn | 32.6819 | 49.0594 | 123.0 | Dfb |
| 70 | 33586 | Zvenygorodka | 30.9000 | 49.0833 | 214.0 | Dfb |
| 71 | 33587 | Uman | 30.2331 | 48.7669 | 214.0 | Dfb |
| 72 | 33446 | Bilopillya | 28.8853 | 49.8403 | 257.0 | Dfb |
| 73 | 33439 | Khmilnyk | 27.9333 | 49.5667 | 284.0 | Dfb |
| 74 | 33562 | Vinnytsya_AMSC | 28.6033 | 49.2481 | 296.0 | Dfb |
| 75 | 33564 | Zhmerynka | 28.1333 | 49.0167 | 313.0 | Dfb |
| 76 | 33577 | Gaysyn | 29.3833 | 48.8000 | 210.0 | Dfb |
| 77 | 33663 | Mogyliv-Podilsky | 27.7833 | 48.4500 | 77.0 | Dfb |
| 78 | 33614 | Svitlovodsk | 33.2497 | 49.0711 | 85.4 | Dfa |
| 79 | 33598 | Novomyrgorod | 31.6489 | 48.8261 | 178.7 | Dfb |
| 80 | 33609 | Znamyanka | 32.6908 | 48.7394 | 180.0 | Dfb |
| 81 | 33711 | Kirovograd_AMSC | 32.2833 | 48.5436 | 170.0 | Dfb |
| 82 | 33686 | Gayvoron | 29.8450 | 48.3447 | 149.5 | Dfb |
| 83 | 33705 | Pomichna | 31.4000 | 48.2333 | 210.7 | Dfb |
| 84 | 33717 | Bobrynets | 32.1667 | 48.0667 | 142.2 | Dfb |
| 85 | 33719 | Dolynska | 32.7506 | 48.1044 | 176.0 | Dfb |
| 86 | 33761 | Lyubashivka | 30.2686 | 47.8508 | 181.0 | Dfb |
| 87 | 33759 | Zatyshshya | 29.8833 | 47.3333 | 193.0 | Dfb |
| 88 | 33833 | Serbka | 30.7519 | 47.0131 | 72.0 | BSk |
| 89 | 33834 | Rozdilna | 30.0708 | 46.8500 | 146.0 | Dfb |
| 90 | 33835 | Odesa_observatory | 30.7703 | 46.4408 | 42.0 | BSk |
| 91 | 33830 | Bilgorod-Dnistrovskyi | 30.3667 | 46.1833 | 1.0 | BSk |
| 92 | 33896 | Sarata | 29.6703 | 46.0242 | 12.0 | BSk |
| 93 | 33887 | Bolgrad | 28.6258 | 45.6828 | 80.0 | BSk |
| 94 | - | Izmail_observatory | 28.8508 | 45.3703 | 28.0 | BSk |
| 95 | 33898 | Vylkove | 29.5928 | 45.3944 | 1.0 | BSk |
| 96 | 33699 | Pervomaysk | 30.8581 | 48.0525 | 104.9 | Dfb |
| 97 | 33777 | Voznesensk | 31.3200 | 47.5825 | 26.1 | Dfa |
| 98 | 33788 | Bashtanka | 32.4681 | 47.4128 | 84.0 | BSk |
| 99 | 33846 | Mykolaiv_AMSC | 31.9094 | 47.0544 | 49.0 | BSk |
| 100 | 33848 | Ochakiv | 31.5389 | 46.6239 | 35.0 | BSk |
| 101 | 33862 | Velyka_Oleksandrivka | 33.2881 | 47.3172 | 57.0 | BSk |
| 102 | 33877 | Nyzhni_Sirogozy | 34.4000 | 46.8500 | 53.5 | BSk |
| 103 | 33869 | Nova_Kakhovka | 33.3586 | 46.7892 | 26.4 | BSk |
| 104 | 33902 | Kherson | 32.7083 | 46.7383 | 47.0 | BSk |
| 105 | 33915 | Askaniya-Nova | 33.8797 | 46.4511 | 28.0 | BSk |
| 106 | 33907 | Bekhtery | 32.2831 | 46.2431 | 6.0 | BSk |
| 107 | 33910 | Genichesk | 34.8072 | 46.1642 | 14.0 | BSk |
| 108 | 33917 | Khorly | 33.3000 | 46.0833 | 6.3 | BSk |
| 109 | 33961 | Strilkove | 34.8778 | 45.9008 | 4.0 | BSk |
| 110 | 33058 | Druzhba | 33.9458 | 52.0531 | 190.0 | Dfb |
| 111 | 33156 | Glukhiv | 33.9833 | 51.6500 | 164.0 | Dfb |
| 112 | 33261 | Konotop | 33.2000 | 51.2333 | 144.0 | Dfb |
| 113 | 33275 | Sumy_AMSC | 34.7481 | 50.8586 | 180.0 | Dfb |
| 114 | 33268 | Romny | 33.4428 | 50.7683 | 168.0 | Dfb |
| 115 | 33382 | Lebedyn | 34.5133 | 50.5872 | 131.0 | Dfb |
| 116 | 33376 | Gadyach | 33.9814 | 50.3669 | 154.0 | Dfb |
| 117 | 33377 | Lubny | 32.9997 | 50.0169 | 155.7 | Dfb |
| 118 | 33495 | Vesely_Podil | 33.2575 | 49.6097 | 96.0 | Dfb |
| 119 | 33506 | Poltava_CHM | 34.5447 | 49.6094 | 160.0 | Dfb |
| 120 | 33621 | Kobelyaky | 34.2078 | 49.1539 | 115.0 | Dfb |
| 121 | 34208 | Zolochiv | 35.9617 | 50.2964 | 159.0 | Dfb |
| 122 | 34302 | Bogodukhiv | 35.4981 | 50.1647 | 196.0 | Dfb |
| 123 | - | Velyky_Burluk | 37.3833 | 50.0667 | 175.0 | Dfb |
| 124 | 34304 | Kolomak | 35.2333 | 49.8500 | 180.0 | Dfb |
| 125 | 34300 | Kharkiv_AMSC | 36.2789 | 49.9267 | 154.0 | Dfb |
| 126 | 34319 | Kupyansk | 37.6500 | 49.6500 | 87.0 | Dfb |
| 127 | 34317 | Komsomolske | 36.5333 | 49.6000 | 102.0 | Dfb |
| 128 | 34401 | Krasnograd | 35.4000 | 49.3500 | 158.0 | Dfb |
| 129 | 34415 | Izyum | 37.3000 | 49.1833 | 77.0 | Dfb |
| 130 | 34409 | Lozova | 36.3167 | 48.9000 | 175.0 | Dfb |
| 131 | 34421 | Svatove | 38.1681 | 49.4100 | 85.0 | Dfb |
| 132 | 34434 | Bilovodsk | 39.5594 | 49.1875 | 74.4 | Dfa |
| 133 | 34523 | Lugansk_CHM | 39.2275 | 48.5656 | 58.8 | Dfa |
| 134 | 34537 | Dariivka | 39.4750 | 48.0711 | 300.0 | Dfa |
| 135 | 34510 | Artemivsk | 38.0147 | 48.5572 | 123.0 | Dfa |
| 136 | 34524 | Debaltseve | 38.4361 | 48.3597 | 334.0 | Dfb |
| 137 | 34514 | Krasnoarmiysk | 37.1561 | 48.2928 | 193.4 | Dfb |
| 138 | 34519 | Donetsk_AMSC | 37.7264 | 48.0722 | 224.0 | Dfb |
| 139 | - | Amvrosiivka | 38.4969 | 47.7922 | 164.0 | Dfa |
| 140 | 34615 | Volnovakha | 37.4833 | 47.6167 | 266.0 | Dfa |
| 141 | 34712 | Mariupol | 37.4844 | 47.0425 | 68.0 | Dfa |
| 142 | 34407 | Gubynykha | 35.2500 | 48.8000 | 127.0 | Dfa |
| 143 | 34504 | Dnipropetrovsk_AMSC | 35.0850 | 48.3600 | 142.0 | Dfa |
| 144 | 34502 | Pavlograd | 35.8878 | 48.5531 | 65.0 | Dfa |
| 145 | 33723 | Komisarivka | 33.9000 | 48.4333 | 118.0 | Dfa |
| 146 | 34505 | Synelnykove | 35.4500 | 48.3500 | 147.0 | Dfa |
| 147 | 34509 | Chaplyne | 36.2378 | 48.1436 | 173.0 | Dfa |
| 148 | 33791 | Kryvyi_Rih_AMSC | 33.2111 | 48.0533 | 123.0 | Dfa |
| 149 | 33801 | Loshkarivka | 34.1733 | 47.9711 | 83.0 | Dfa |
| 150 | 33805 | Nikopol | 34.4019 | 47.5922 | 53.0 | BSk |
| 151 | 34601 | Zaporizhzhya_CHM | 35.0825 | 47.8789 | 59.3 | Dfa |
| 152 | 34606 | Gulyaypole | 36.2683 | 47.6503 | 117.0 | Dfa |
| 153 | 34607 | Pryshyb | 35.3325 | 47.2606 | 86.8 | Dfa |
| 154 | 34609 | Kyrylivka | 36.3344 | 47.3303 | 220.9 | Dfa |
| 155 | 34704 | Melitopol | 35.3564 | 46.8303 | 33.3 | BSk |
| 156 | 34717 | Berdyansk_port | 36.7833 | 46.7500 | 1.0 | BSk |
| 157 | 34708 | Botieve | 35.8431 | 46.6833 | 17.0 | BSk |
| 158 | 33933 | Ishun | 33.8161 | 45.9269 | 3.0 | BSk |
| 159 | 33934 | Dzhankoy | 34.3997 | 45.7042 | 6.0 | BSk |
| 160 | 33924 | Chornomorske | 32.7161 | 45.5217 | 9.0 | BSk |
| 161 | 33962 | Nyzhnogirskyi | 34.7097 | 45.4467 | 19.0 | BSk |
| 162 | 33939 | Klepinine | 34.1756 | 45.5261 | 37.0 | BSk |
| 163 | 33981 | Mysove | 35.8228 | 45.4497 | 15.0 | BSk |
| 164 | 33983 | Kerch | 36.4147 | 45.3742 | 46.0 | BSk |
| 165 | 33986 | Opasne | 36.6333 | 45.3667 | 0.0 | BSk |
| 166 | 33929 | Evpatoriya_port | 33.3744 | 45.1897 | 2.0 | BSk |
| 167 | 33973 | Vladyslavivka | 35.3797 | 45.1706 | 35.0 | BSk |
| 168 | 33966 | Bilogirsk | 34.5986 | 45.0453 | 205.0 | Cfa |
| 169 | 33946 | Simferopol_AMSC | 33.9672 | 45.0403 | 180.0 | BSk |
| 170 | 33976 | Feodosiya | 35.3817 | 45.0372 | 22.0 | BSk |
| 171 | 33945 | Poshtove | 33.9436 | 44.8325 | 172.0 | BSk |
| 172 | - | Angarsky_pereval | 34.3411 | 44.7389 | 765.0 | Dfb |
| 173 | 33959 | Alushta | 34.4022 | 44.6606 | 70.0 | Cfa |
| 174 | - | Khersones_mayak | 33.3822 | 44.5831 | 2.0 | BSk |
| 175 | 33991 | Sevastopol | 33.5325 | 44.6169 | 7.0 | BSk |
| 176 | 33998 | Ay-Petri | 34.0686 | 44.4692 | 1180.0 | Dfb |
| 177 | 33990 | Yalta | 34.1553 | 44.4811 | 66.0 | Cfa |
| 178 | - | Nikitsky_Sad | 34.2403 | 44.5128 | 207.0 | Cfa |
| Hydrological posts | | | | | | |
| 1 | - | Richytsya | 24.6575 | 51.7589 | 154.0 | Dfb |
| 2 | - | Vyzhva | 24.4117 | 51.3997 | 168.0 | Dfb |
| 3 | - | Ruda | 24.2508 | 51.2292 | 184.0 | Dfb |
| 4 | - | Yagidne | 24.3261 | 51.0278 | 186.0 | Dfb |
| 5 | - | Svaryni | 26.2544 | 51.2786 | 161.0 | Dfb |
| 6 | - | Derazhne | 26.0494 | 50.8622 | 174.0 | Dfb |
| 7 | - | Schurovychi | 25.0217 | 50.2675 | 195.0 | Dfb |
| 8 | - | Trijtsya | 24.7656 | 50.1486 | 207.0 | Dfb |
| 9 | - | Busk | 24.6083 | 49.9683 | 220.0 | Dfb |
| 10 | - | Sasiv | 24.9489 | 49.8708 | 271.0 | Dfb |
| 11 | - | Zhvanets | 26.4875 | 48.5500 | 158.0 | Dfb |
| 12 | - | Pidgajtsi | 25.8864 | 49.9750 | 346.0 | Dfb |
| 13 | - | Galych | 24.7317 | 49.1394 | 217.0 | Dfb |
| 14 | - | Dora | 24.5786 | 48.4758 | 489.0 | Dfb |
| 15 | - | Litky | 30.7461 | 50.7028 | 102.0 | Dfb |
| 16 | - | Berezan | 31.4850 | 50.3075 | 101.0 | Dfb |
| 17 | - | Zdorivka | 30.2492 | 50.1919 | 149.0 | Dfb |
| 18 | - | Fesyury | 29.9644 | 49.6611 | 181.0 | Dfb |
| 19 | - | Korsun-Shevchenkivskyi | 31.2772 | 49.4194 | 104.0 | Dfb |
| 20 | - | Lysyanka | 30.8233 | 49.2569 | 145.0 | Dfb |
| 21 | - | Yampil | 30.9781 | 48.7681 | 116.0 | Dfb |
| 22 | - | Krupoderyntsi | 29.3394 | 49.5053 | 203.0 | Dfb |
| 23 | - | Lityn | 28.0833 | 49.3272 | 263.0 | Dfb |
| 24 | - | Zoziv | 29.0139 | 49.3175 | 246.0 | Dfb |
| 25 | - | Oleksandro-Stepanivka | 33.1519 | 48.6083 | 99.0 | Dfb |
| 26 | - | Pokotylove | 30.6928 | 48.4725 | 118.0 | Dfb |
| 27 | - | Osychky | 30.0289 | 48.1311 | 98.0 | Dfb |
| 28 | - | Berezivka | 30.9172 | 47.2006 | 19.0 | BSk |
| 29 | - | Kuyalnytskyj_lyman | 30.7131 | 46.6644 | 1.0 | BSk |
| 30 | - | Reni | 28.2711 | 45.4575 | 5.0 | BSk |
| 31 | - | Kiliya | 29.2378 | 45.4675 | 4.0 | BSk |
| 32 | - | Chongarskyj_Mist | 34.5458 | 46.0308 | 9.0 | BSk |
| 33 | - | Rozdolne | 33.4833 | 45.7833 | 16.0 | BSk |
| 34 | - | Oleksijevo-Druzhkivka | 37.6147 | 48.5806 | 75.0 | Dfa |
| 35 | - | Prykolotne | 37.3500 | 50.1667 | 213.0 | Dfb |
| 36 | - | Oleksijevo-Orlivka | 38.5886 | 48.1800 | 126.0 | Dfb |
| 37 | - | Verkhnodniprovsk | 34.3403 | 48.6425 | 90.0 | Dfa |
| 38 | - | Kokhanivka | 36.4375 | 48.5197 | 92.0 | Dfa |
| 39 | - | Krynychky | 34.4564 | 48.3736 | 79.0 | Dfa |
| 40 | - | Vasylkivka | 36.0253 | 48.2083 | 76.0 | Dfa |
| 41 | - | Pology | 36.2761 | 47.4756 | 94.0 | Dfa |
| 42 | - | Dvorichchya | 34.8022 | 45.4844 | 14.0 | BSk |
| 43 | - | Zarichchya | 34.7097 | 45.3625 | 39.0 | BSk |
| 44 | - | Zybyny | 34.6536 | 45.2350 | 78.0 | BSk |
| 45 | - | Topolivka | 34.8789 | 45.0106 | 253.0 | Dfb |
| 46 | - | Mizhgirjya_Krym | 34.4053 | 44.9828 | 378.0 | Dfb |
| 47 | - | Sudak | 34.9667 | 44.8500 | 24.0 | BSk |
| 48 | - | Fruktove | 33.6031 | 44.6806 | 17.0 | BSk |
| 49 | - | Bashtanivka | 33.8947 | 44.6886 | 173.0 | Dfa |
| 50 | - | Rodnykivske | 33.8472 | 44.4625 | 269.0 | Dfb |
| Comments. AMSC means Aviation Meteorological Station Civil; CHM stands for Center for Hydrometeorology | | | | | | |
